# Estimation of activity of administered ^18^F-fluorodeoxyglucose by measurement of the dose equivalent rate on the right temporal region of the head

**DOI:** 10.1186/s40658-016-0164-1

**Published:** 2016-11-14

**Authors:** Kenta Sakaguchi, Makoto Hosono, Tomomi Imamura, Naomi Takahara, Misa Hayashi, Yuko Yakushiji, Kazunari Ishii, Tatsuro Uto, Takamichi Murakami

**Affiliations:** 1 Division of Positron Emission Tomography, Institute of Advanced Clinical Medicine, Faculty of Medicine, Kindai University, 377-2 Ohno-Higashi, Osaka-Sayama, 589-8511 Osaka Japan; 2 Department of Radiology, Faculty of Medicine, Kindai University, Osaka-Sayama, Japan; 3 Division of Nursing, Kindai University Hospital, Osaka-Sayama, Japan

## Abstract

**Background:**

Positron emission tomography (PET) with ^18^F-fluorodeoxyglucose (FDG) is now a routine procedure for the management of cancer patients. Intravenous administration of FDG is sometimes halted due to troubles. In such cases, estimations of the FDG dosage injected prior to halting administration may be helpful. We have established a method of estimating the activity of FDG to patients on the basis of the dose equivalent rate on the surface of the right temporal region of the head. The correlation of actual administered dosage with independent variables, such as the dose equivalent rate on the right temporal region of the head, age, sex, and body weight, was analyzed using multiple regression analysis to obtain linear, quadratic, and cubic regression equations.

**Results:**

When entering independent variables, the cubic regression equation could be used to estimate an administered dosage with an accuracy of ±10 % for 62 % of all patients and ±20 % for 90 % of all patients.

**Conclusions:**

We conclude that this method is useful for estimating the administered dosage from the dose equivalent rate on the temporal region of the head.

## Background

Exploiting the enhanced glucose uptake by tumor cells as compared with normal cells, positron emission tomography (PET) with ^18^F-fluorodeoxyglucose (FDG) is a routine procedure for the management of cancer patients [1–3]. Intravenous administration of FDG is sometimes halted due to extravasation of FDG, pain, or malfunction of injection devices. In such cases, estimations of the FDG dosage injected prior to halting administration may be helpful in making a decision on how to proceed, whether to perform venipuncture at a different site to inject additional FDG or to estimate the eventual influence of underdosing on standardized uptake values (SUVs) and image quality [4–6]. Thus, the activity remaining in the line and syringe may be estimated by using a radionuclide calibrator. This, however, may increase unnecessary radiation exposure to staffers because they have to handle the line and syringe and sometimes the injection device which may contain a large amount of radioactivity. To date, few studies have attempted to establish methods for estimating the administered dosage against such incomplete dosages immediately after administration was halted [7]. In this study, the dose equivalent rate on the head of patients was assumed to give accurate estimates of administered dosages. The aim of this study was to establish a method for estimating the administered dosage based on variables including, dose equivalent rate on the right temporal region of the head, age, sex, body weight (weight), height, elapsed time from administration, and blood sugar using multiple regression analysis.

## Methods

### Patients and data collection

Seven hundred six patients underwent PET examination in our site during the period between the 9th of March 2015 and the 23rd of May 2015. Of these, 520 patients were prospectively enrolled in this study with Institutional Review Board approval. The requirement to obtain informed consent was waived. The remaining 180 patients were not included in this study because of refusal to be involved, unfavorable physical condition, or incomplete records.

Dosage calculation in our site:

1 $$ \mathrm{Administered}\;\mathrm{Dosage}\;\left[\mathrm{MBq}\right]=\mathrm{I}\mathrm{n}\mathrm{t}\left(\frac{\mathrm{Weight}\left[\mathrm{kg}\right]}{10}\right)\times 10\times 3\left[\mathrm{MBq}/\mathrm{kg}\right] $$

where Int() is the function of “round down to the nearest decimal.” For example, for a 69-kg patient, 180 MBq is administered using an FDG automated injector (M130, SUMITOMO Heavy Industries, Tokyo, Japan). After FDG administration, using an FDG automated injector, the dose equivalent rate on the right temporal lobe (DER) was measured with the patient sitting upright using an ionization chamber type survey meter (ICS-323, HITACHI ALOKA). No patients voided their bladders between injection and dose-rate measurement. The right temporal lobe was chosen based on patient comfort and the ability to conduct stable measurements. DER, administered dosage, and elapsed time following FDG administration were recorded, as well as age, sex, height, weight, and blood sugar. There are two reasons for having adopted the elapsed time following FDG administration as a dependent variable, the first is to reflect the physical decay and the second is to follow time-dependent uptake of the tracer in the brain. All data were recorded by six nurses working in our PET institution.

### Multiple regression analysis

This study was performed on a presupposition that DER should be in proportion to AD. However, DER/AD (i.e., DER per unit dosage) is not completely a constant value. DER/AD is influenced by patients’ characteristics, such as sex, elapsed time following administration, patient age, height, weight, and blood sugar. Therefore, to achieve the presuppositions, DER had to be converted to corrected DER (CDER), which was unaffected by the influence of patients’ characteristics. The process of obtaining CDER is demonstrated as follows. IBM SPSS statistics (International Business Machines Corporation) was employed for analysis and calculation of multiple regression analysis.

**Fig. 1:**
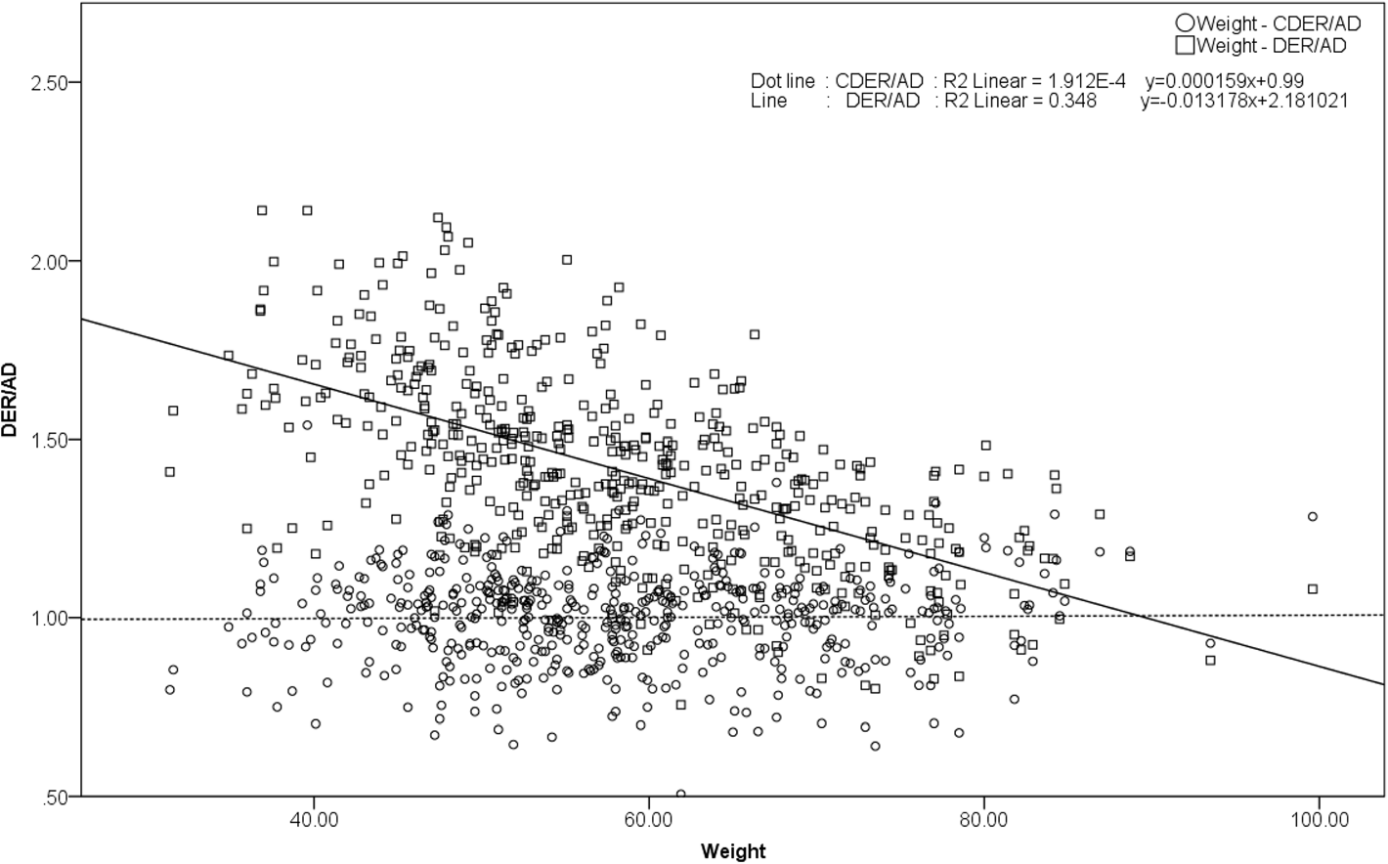
Scatter plot representing DER/AD. *DER* dose equivalent rate/administered dosage, *AD* administered dosage, *CDER* corrected DER

Figure 1 shows the scatter plot representing DER/administered dosage (DER/AD; squares) on the vertical axis and weight on the horizontal axis. The DER/AD plot can be used to determine a constant coefficient for each patient. In this plot, DER/AD decreases as weight increases. A correction coefficient can be calculated using the single regression equation and the DER/AD and weight plots.

2 $$ \mathrm{Correction}\ \mathrm{Coefficient}=-0.013178\times \mathrm{weight}+2.181021 $$

And, corrected DER (CDER) is given by the equation:

3 $$ \mathrm{CDER}=\frac{1}{\mathrm{Correction}\;\mathrm{Coefficient}}\times \mathrm{D}\mathrm{E}\mathrm{R}=\frac{1}{-0.013178\times \mathrm{weight}+2.181021}\times \mathrm{D}\mathrm{E}\mathrm{R} $$

In Fig. 1, the plotted circles show no significant correlation between CDER/AD and weight, demonstrating that the corrected DER is independent of weight. On the basis of the above procedure of obtaining single regression analysis, the correction coefficient for DER was calculated by stepwise multiple regression analysis with the dependent variable DER/AD and independent variables of elapsed time, age, sex (dummy variables: male = 1, female = 0), blood sugar, and either height or weight or body mass index (BMI). CDER was calculated using the equation:

4 $$ \mathrm{CDER}=\frac{1}{\mathrm{Correction}\;\mathrm{Coefficient}\;\mathrm{with}\;\mathrm{Stepwise}\;\mathrm{Multiple}\;\mathrm{R}\mathrm{egression}\ \mathrm{E}\mathrm{quation}}\times \mathrm{D}\mathrm{E}\mathrm{R} $$

Here, it should be noted that weight, height, and BMI exhibit multicollinearity. Therefore, the most appropriate variable, of the three, was determined using the Pearson test. The variable with the highest Pearson correlation coefficient was determined to be the most important independent variable.

Next, an estimation of the administered dosage was calculated using CDER by linear, quadratic, and cubic regression analyses.

5 $$ \mathrm{Linear}:\kern0.37em \mathrm{Estimated}\ \mathrm{Administered}\;\mathrm{Dosage}=L(1)\times \mathrm{CDER} $$

6 $$ \mathrm{Quadratic}:\ \mathrm{E}\mathrm{stimated}\ \mathrm{Administered}\ \mathrm{D}\mathrm{osage} = Q(1)\times \mathrm{C}\mathrm{D}\mathrm{E}{\mathrm{R}}^2+Q(2)\times \mathrm{CD}\mathrm{E}\mathrm{R} $$

7 $$ \mathrm{C}\mathrm{ubic}:\kern0.24em \mathrm{E}\mathrm{stimated}\;\mathrm{Administered}\ \mathrm{D}\mathrm{osage}=C(1)\times \mathrm{C}\mathrm{D}\mathrm{E}{\mathrm{R}}^3+C(2)\times \mathrm{C}\mathrm{D}\mathrm{E}{\mathrm{R}}^2+C(3)\times \mathrm{C}\mathrm{D}\mathrm{E}\mathrm{R} $$

where *L*(1), *Q*(1), *Q*(2), *C*(1), *C*(2), and *C*(3) are regression coefficients, and the intercept is zero.

Finally, the accuracy (residual rate) of the regression equations was calculated.

8 $$ \mathrm{Residual}\;\mathrm{Rate}=\frac{\mathrm{Estimated}\;\mathrm{Administered}\;\mathrm{Dose}\ \left[\mathrm{M} Bq\right]}{\mathrm{Administered}\;\mathrm{Dose}\ \left[ MBq\right]}-1 $$

## Results

### Test of normality

There were no significant differences in DER/AD among the nurses (data now shown). Descriptive statistics and tests of normality, with the Shapiro-Wilk test results for each of the dependent and independent variables, are shown in Tables 1 and 2. Table 2 shows the Shapiro-Wilk test *p* values for DER, DER/AD, height, and others variables. Because the *p* values for DER and DER/AD were higher than 0.05, the null hypothesis was not rejected, indicating that the DER and DER/AD data were normally distributed and that the independent variables were appropriate for calculation of CDER with stepwise multiple regression analysis.

**Table 1:** Variables and statistics

Variables	Mean ± SD	Range	Skewness	Kurtosis
Administered dose [MBq]	152.7 ± 35.9	73.8–281.2	0.458	0.181
Elapsed time [s]	217.0 ± 95.1	36.0–620.0	0.001	−0.720
Dose equivalent rate [μSv/h]	210.6 ± 41.0	104.0–374.0	0.220	0.191
Height [cm]	160.7 ± 9.4	129.0–185.0	0.035	−0.376
Weight [kg]	58.12 ± 11.6	31.4–99.6	0.382	−0.185
Blood sugar [mg/dl]	112.6 ± 24.6	60.0–274.0	2.599	10.431
Age [years]	66.1 ± 12.2	18.0–91.0	−0.863	0.873
DER/AD [(μSv/h)/MBq]	1.41 ± 0.26	0.76–2.14	0.226	−0.103

Sex: male = 286; female = 234

*DER/AD* dose equivalent rate per administered dose

**Table 2:** Shapiro-Wilk test of variables

	Shapiro-Wilk test	
	*W*	*P* value
Administered dose [MBq]	0.980	0.000
Elapsed Time [s]	0.878	0.000
Dose equivalent rate [μSv/h]	0.995	0.096
Height [cm]	0.993	0.020
Weight [kg]	0.987	0.000
Blood sugar [mg/dl]	0.780	0.000
Age [years]	0.955	0.000
DER/AD [(μSv/h)/MBq]	0.995	0.062

*DER/AD* dose equivalent rate per administered dose

### Calculation of CDER with stepwise multiple regression analysis

The bivariate Pearson correlation coefficients for height, weight, and BMI (−0.452, −0.590, and −0.414, respectively) are shown in Table 3. Because the Pearson correlation was highest for weight, we adopted elapsed time, age, sex, blood sugar, and weight as independent variables and performed stepwise regression analysis with these independent variables and the dependent variable CDER/AD.

**Table 3:** DER/AD and height, weight, and BMI

		Height	Weight	BMI
DER/AD	Pearson correlation	−0.452	−0.590	−0.414
	*p* value (2-tailed)	0.000^a^	0.000^a^	0.000^a^

DER/AD dose equivalent rate per administered dose

^a^Correlation is significant at the 0.01 level (2-tailed)

Table 4 shows the results of stepwise multiple regression analysis. The significance of each independent variable was less than 0.01, indicating no influence, in all models, of the independent variables on the dependent variable. All variance inflation factors (VIFs) were less than 1.30, indicating no multicollinearity. Therefore, model 4 was used because it produced the highest adjusted *R*-square.

**Table 4:** Stepwise multiple regression analysis models

Model		Adjusted *R*-square	Unstandardized coefficients		Standardized coefficients	*t* value	Sig.	Collinearity statistics	
			Regression coefficient	Std. error	Beta			Tolerance	VIF
1	(Constant)	0.346	2.181021	0.047049		46.356	0.000		
	Weight [kg]		−0.013178	0.000793	−0.590	−16.614	0.000	1.000	1.000
2	(Constant)	0.414	2.022751	0.048944		41.327	0.000		
	Weight [kg]		−0.013117	0.000751	−0.587	−17.465	0.000	1.000	1.000
	Elapsed time [s]		0.000713	0.000091	0.262	7.804	0.000	1.000	1.000
3	(Constant)	0.451	2.237429	0.059558		37.567	0.000		
	Weight [kg]		−0.012969	0.000728	−0.580	−17.823	0.000	0.999	1.001
	Elapsed Time [s]		0.000743	0.000089	0.273	8.385	0.000	0.997	1.003
	Blood sugar [mg/dl]		−0.002040	0.000343	−0.194	−5.951	0.000	0.996	1.004
4	(Constant)	0.458	2.197180	0.060822		36.124	0.000		
	Weight [kg]		−0.011904	0.000814	−0.533	−14.626	0.000	0.787	1.270
	Elapsed time [s]		0.000752	0.000088	0.277	8.542	0.000	0.995	1.005
	Blood Sugar [mg/dl]		−0.001987	0.000341	−0.189	−5.829	0.000	0.993	1.007
	Sex		−0.053893	0.018947	−0.104	−2.844	0.005	0.784	1.275

*VIF* variance inflation factor

9 $$ \begin{array}{l}\mathrm{Corrected}\;\mathrm{Coefficient}\\ {}\kern3.48em =-0.053893\times \mathrm{Sex}+0.000752\times \mathrm{Elapsed}\;\mathrm{Time}\hbox{-} 0.011904\times \mathrm{Weight}\\ {}\kern3.96em -0.001987\times \mathrm{Blood}\;\mathrm{Sugar}+2.197180\end{array} $$

10 $$ \mathrm{CDER}=\frac{1}{-0.053893\times \mathrm{Sex}+0.000752\times \mathrm{Elapsed}\;\mathrm{Time}\hbox{-} 0.011904\times \mathrm{Weight}\hbox{-} 0.001987\times \mathrm{Blood}\;\mathrm{Sugar}+2.197180}\times \mathrm{D}\mathrm{E}\mathrm{R} $$

**Fig. 2:**
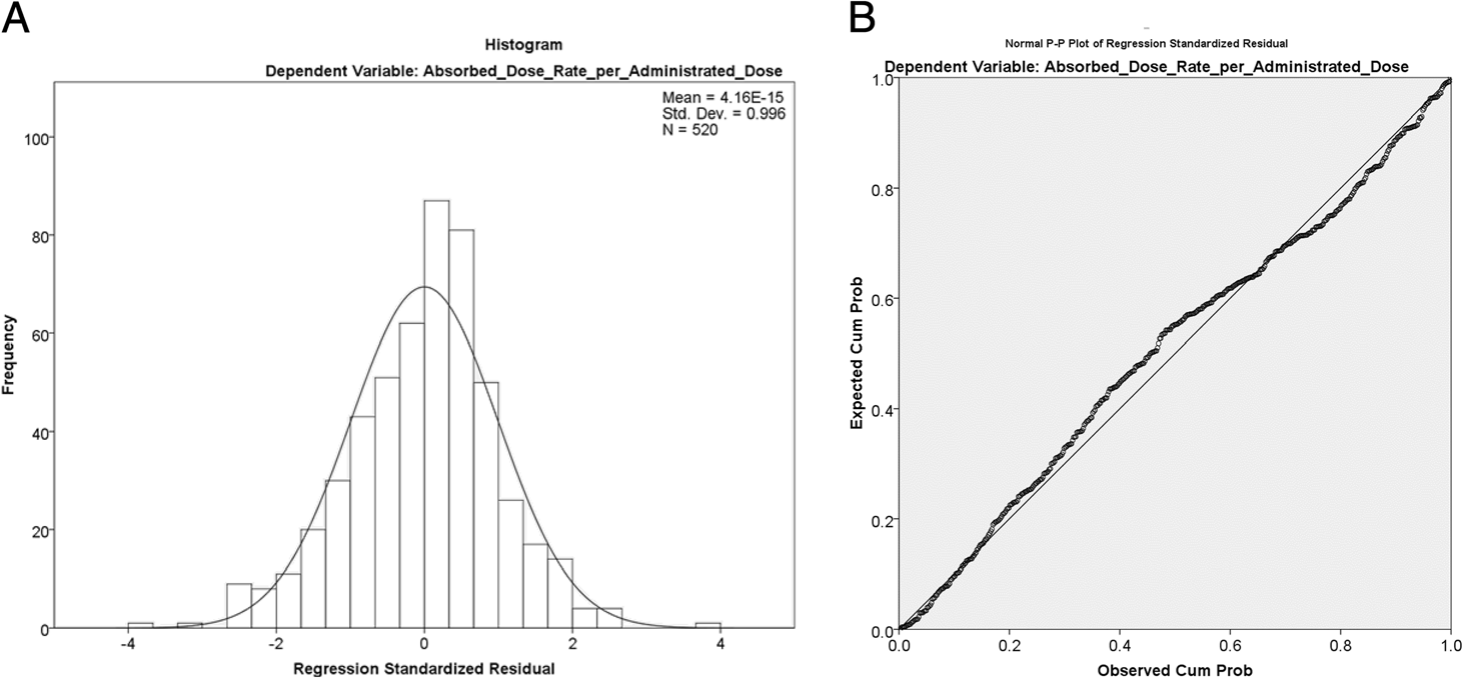
Regression standardized residuals as normal P-P plot of **a** and histogram **b**

Figure 2 shows the normal P-P plot (a) and histogram of regression standardized residuals (b). In Fig. 2a, plotted points deviating greatly from a straight line indicate that the normality assumption was not met. The plotted points showed a good agreement with the straight line. Figure 2b shows that the histograms were normally distributed around zero. Moreover, the mean of residual rates was nearly zero. These results indicate that the assumption of a normal distribution of residuals was satisfied.

### Estimation of administered dosage with linear and nonlinear regression analysis

**Fig. 3:**
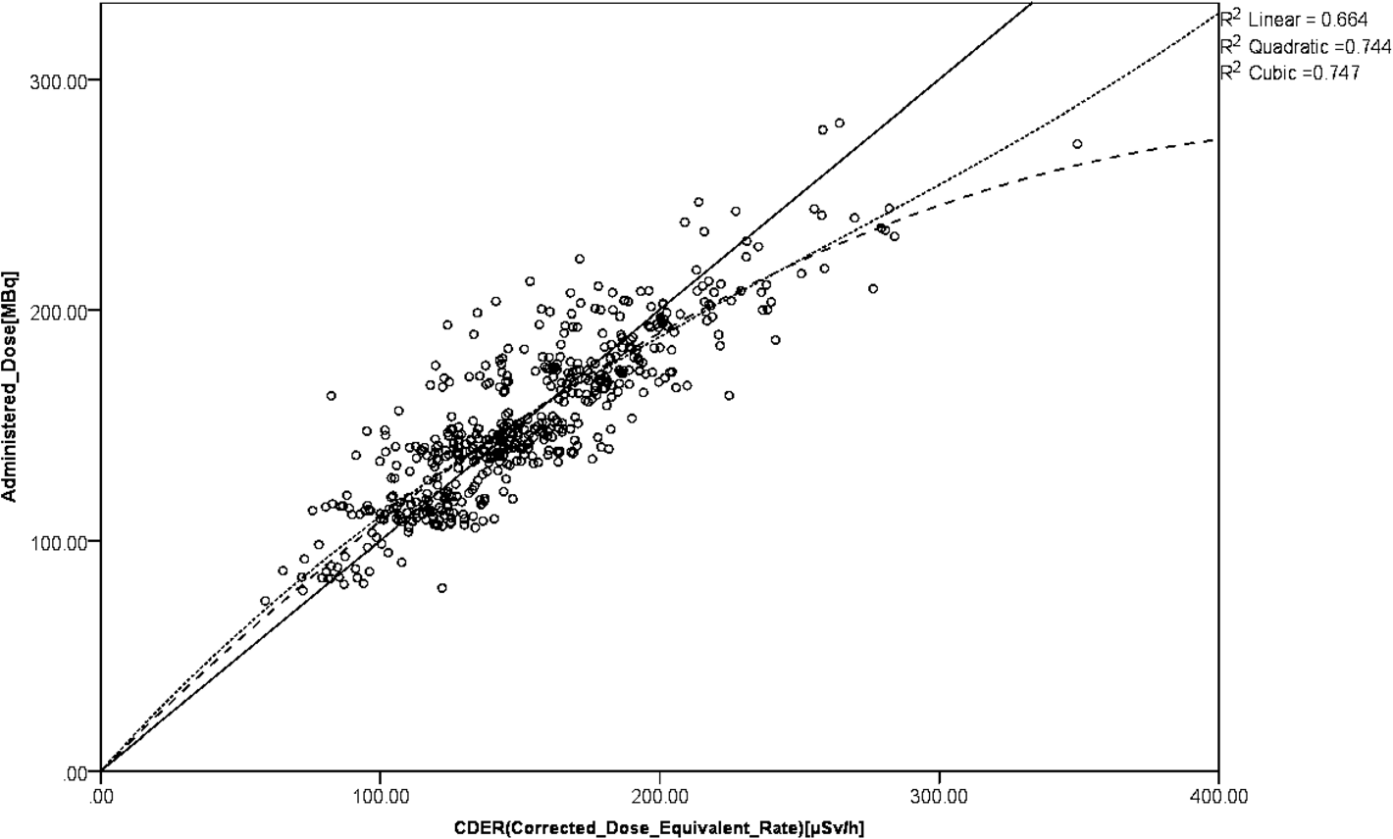
Scatter plot with CDER and administered dosage. *Solid line*, linear; *long dashed line*, quadratic; *short dashed line*, cubic

Figure 3 shows the scatter plot with CDER on the *X*-axis and administered dosage on the *Y*-axis. Table 5 shows estimated parameters with linear, quadratic, and cubic regression equations for estimating the administered dosage. The adjusted *R*-square for linear, quadratic, and cubic equations is 0.664 (moderate correlation), 0.744 (strong correlation), and 0.747 (strong correlation), respectively.

**Table 5:** Parameter estimates

Parameter	Estimate	Standard error	95% confidence interval	
			Lower bound	Upper bound
L1	9.832E−001	5.747E−003	9.719E−001	9.945E−001
Q1	−1.328E−003	0.000E+000	−1.328E−003	−1.328E−003
Q2	1.216E+000	1.081E−004	1.216E+000	1.217E+000
C1	3.422E−006	1.491E−006	4.926E−007	6.352E−006
C2	−2.654E−003	5.878E−004	−3.809E−003	−1.499E−003
C3	1.336E+000	5.578E−002	1.227E+000	1.446E+000

**Fig. 4:**
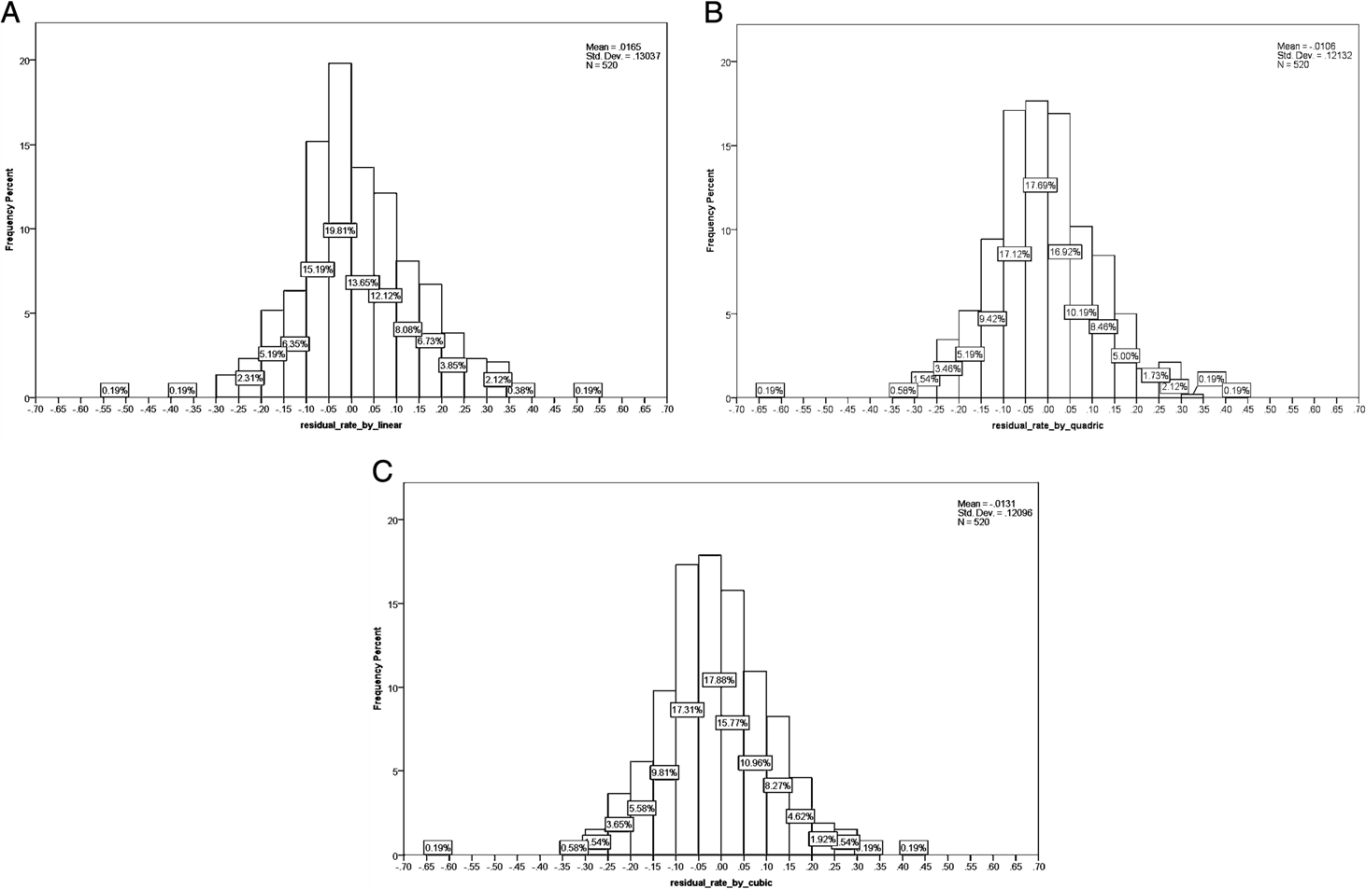
Residual rate histograms: linear **a**, quadratic **b**, and cubic **c** estimations

Figure 4 shows the residual rates of histograms calculated by linear, quadratic, and cubic estimations. These histograms showed that the residual rates were normally distributed around zero, and the mean of the residual rates was nearly zero. Using linear, quadratic, and cubic regression equations, respectively, the administered dosage in 60.77 % (316/520), 61.92 % (322/520), and 61.92 % (322/520) of the patients could be estimated with an accuracy of ±10 %. The above population percentages have been found to be 87.12 % (453/520), 90.00 % (468/520), and 90.20 % (469/520), respectively, for an estimation accuracy of ±20 %.

## Discussion

We have described a method to estimate the actual dosage of FDG administered using corrected DER and four independent variables of sex, weight, blood sugar, and elapsed time following administration in a cubic regression equation obtained by analyzing 520 patients. The ratio of DER over actual administered dosage becomes practically constant when we correct DER using the independent variables, as indicated, by stepwise multiple regression analysis. This method is useful for calculating the radioactivity of FDG administered to patients when administration must be halted for any reason. However, it does not necessarily give a precise value of administered dosage but just an estimate. Care should be taken when an administered dosage is estimated by our method and it may affect the diagnostic interpretation or quantification of FDG accumulation. Of the four independent variables, the influence of blood sugar was less than weight and elapsed time following administration in stepwise multiple regression analysis. This is interesting because usually blood sugar affects brain uptake of FDG [8, 9]. One proposed reason for the decreased influence, of blood sugar on FDG uptake in the brain, might be that DER was measured shortly after administration of FDG. Thus, the effect of blood sugar on brain uptake was not large [10].

Because the proposed estimation method makes use of dose equivalent rates on the surface of the right temporal lobe, other factors, that may affect brain uptake of FDG and, consequently, the accuracy of the method, should be considered. Diversion of FDG to large tumors, leading to decreased brain uptake, has already been reported in malignancies including lymphoma [11]. In this study, FDG uptake into tumors was not included as a variable because routinely tumor uptake is unknown at the very time of administration of FDG; thus, it cannot be incorporated in the estimation of the administered dosage.

One limitation of this study is that incomplete injection of FDG, in patients, was not examined, which means that the in vivo predictive power of this estimation method has not been verified. Of particular concern is the case of extravasation, where measurement of the actual radioactivity of FDG injected into the circulation is difficult to determine because FDG could be absorbed by subcutaneous tissue or vascular tissue. DER is most likely associated with radioactivity injected into the circulation. In time, the accuracy of our proposed estimation method can be verified using data accumulated from cases in which DER, the independent variables, and quantitative image data are all known. Another limitation is that this study was conducted at only one facility. To establish such regression approaches, including populations from multiple sites may be crucial to enhance generic applicability and wide clinical adoption. Further, multi-center studies are needed to determine the viability of our estimation method in PET facilities with various clinical circumstances.

## Conclusions

The actual administered dosage of FDG in patients was successfully estimated on the basis of dose the equivalent rate using multiple regression analysis with four independent variables of sex, weight, blood sugar, and elapsed time following administration. We propose using this method to determine the radioactivity of FDG administered to patients in cases of incomplete injection.
